# Association of gastric inhibitory polypeptide receptor (GIPR) gene polymorphism with type 2 diabetes mellitus in iranian patients

**DOI:** 10.1186/s12920-023-01477-z

**Published:** 2023-03-07

**Authors:** Saiedeh Erfanian, Hamed Mir, Amir Abdoli, Abazar Roustazadeh

**Affiliations:** 1 Department of Biochemistry and Nutrition, Jahrom University of Medical Sciences, Jahrom, Iran; 2 Department of Advanced Medical Sciences and Technologies, Jahrom University of Medical Sciences, Jahrom, Iran; 3 Department of Parasitology, School of medicine, Jahrom University of Medical Sciences, Jahrom, Iran; 4 Research Center for Non-Communicable Diseases, Jahrom University of Medical Sciences, Jahrom, Iran; 5 Ostad motahhari Blvd, Jahrom University of Medical Sciences, 74148-46199 Jahrom, Iran

**Keywords:** Gastric inhibitory polypeptide receptor, Polymorphism, Type 2 diabetes mellitus, Middle East

## Abstract

**Introduction:**

Gastric inhibitory polypeptide receptor (GIPR) encodes a G-protein coupled receptor for gastric inhibitory polypeptide (GIP), which was demonstrated to stimulate insulin secretion. Relation of GIPR gene variation to impaired insulin response has been suggested in previous studies. However, little information is available regarding GIPR polymorphisms and type 2 diabetes mellitus (T2DM). Hence, the aim of the study was to investigate single nucleotide polymorphisms (SNPs) in the promoter and coding regions of GIPR in Iranian T2DM patients.

**Materials and methods:**

Two hundred subjects including 100 healthy and 100 T2DM patients were recruited in the study. Genotypes and allele frequency of rs34125392, rs4380143 and rs1800437 in the promoter, 5ʹ UTR and coding region of GIPR were investigated by RFLP-PCR and Nested-PCR.

**Results:**

Our finding indicated that rs34125392 genotype distribution was statistically different between T2DM and healthy groups (P = 0.043). In addition, distribution of T/- + -/- versus TT was significantly different between the both groups (P = 0.021). Moreover, rs34125392 T/- genotype increased the risk of T2DM (OR = 2.68, 95%CI = 1.203–5.653, P = 0.015). However, allele frequency and genotype distributions of rs4380143 and rs1800437 were not statistically different between the groups (P > 0.05). Multivariate analysis showed that the tested polymorphisms had no effect on biochemical variables.

**Conclusion:**

We concluded that GIPR gene polymorphism is associated with T2DM. In addition; rs34125392 heterozygote genotype may increase the risk of T2DM. More studies with large sample size in other populations are recommended to show the ethnical relation of these polymorphisms to T2DM.

**Supplementary Information:**

The online version contains supplementary material available at 10.1186/s12920-023-01477-z.

## Introduction

Global studies indicated that 415 million people lived with diabetes mellitus (DM) until 2017. Estimations suggest that 608 million people are suffered from DM that 32% of them are undiagnosed. More than 90% of DM patients accounts for type 2 diabetes mellitus (T2DM) [1]. T2DM is a common metabolic disorders mainly caused by two mechanisms: reduced insulin secretion by pancreatic B cells or resistance of target tissues to insulin [2]. Any defect in one of these mechanisms could trigger a T2DM phenotype. The most popular risk factors for T2DM are combinations of genetic, environmental and metabolic factors [3, 4] Contribution of genes to T2DM is well studied previously and large-scale genotyping using the Metabochip indicated that some loci increase the susceptibility to T2DM [5]. Now the question is that whether the risk loci are confined to the ones that have been identified in previous studies?

Gastric inhibitory polypeptide receptor (GIPR, Gene ID: 2696) or glucose-dependent insulinotropic polypeptide receptor (https://www.uniprot.org/uniprotkb/P48546/entry) has several isoforms and encodes a G-protein coupled receptor for gastric inhibitory polypeptide (GIP), which was originally identified as a hormone that acts in gut extracts (https://www.ncbi.nlm.nih.gov/gene/2696) and inhibits the releasing of gastrin and subsequently gastric acid. GIPRs are located in pancreas and insulin-sensitive tissues such as adipocytes [6] and their interaction with GIP led to increases of lipoprotein lipase activity (LPL), fatty acid and glucose uptake. Recent studies demonstrated that GIP is secreted in response to oral glucose and stimulates insulin release [7]. Moreover, GIP induces fatty acid adsorption into adipocytes and inhibits lipolysis [8], induces resistance to insulin in adipose tissues [9–11], and the plasma level of GIP is increased in T2DM [12, 13].

GIPR gene located on 19q13.32 is present in β cells of Langerhans islands. Despite differential expression in extra pancreatic tissues, its expression in adipose tissues is relatively high [6]. Previous findings suggest that pharmacological activation of GIPR may have a therapeutic benefit on peripheral energy metabolism [14].

As indicated by recent studies, single nucleotide polymorphism in GIPR gene are related to changes in the secretion of hormones and adipokines in obese type 2 diabetic patients [15]. GIPR gene polymorphisms have been studied in other diseases including schizophrenia [16, 17], pre-diabetic and diabetic patients [18] metabolic syndrome [19] and obesity [20]. However, little information is available in diabetic patients in Iran. Hence, the aim of the present study was to investigate the association of the rs34125392, rs4380143 and rs1800437 polymorphisms in the promoter, 5ʹ UTR and coding region of GIPR gene with type 2 diabetes mellitus in an Iranian population.

## Materials and methods

### Subjects

Two hundred subjects referred to Peymanieh hospital (Jahrom city, Iran) including 100 healthy individuals and 100 type 2 diabetes mellitus patients were recruited in the study. Patients had a fasting blood glucose (FBG) > 125 mg/dl. However, subjects with underlying disease including cancer, liver and kidney disease, gastrointestinal tract disease and cardiovascular disease such as acute myocardial infarction were excluded from the study. Low density lipoprotein cholesterol (LDL-C), high density lipoprotein cholesterol (HDL-C), total cholesterol (TC), triacylglycerol (TG) and FBG were measured by routine biochemical assays.

### DNA extraction and PCR-RFLP

Venous blood samples were drawn in an EDTA-containing tube and stored in -80 ^C^ until the DNA extraction. Salting out technique was used to extract the genomic DNA [21] and stored rapidly in -20. Three single nucleotide polymorphisms were investigated in the study. On the other hand, rs4380143 T > C with minor allele frequency (MAF) 0.3 was located on upstream promoter region, rs34125392 T>- with MAF 0.26 was located on 5ʹUTR region, and rs1800437 G > C (Glu to Gln) with MAF 0.2 was located on coding region. RFLP-PCR was applied to genotyping and allele identification. The sequences of primers used in the study are summarized in Table 1.

**Table 1:** Sequence of primers used for detection of rs34125392, rs4380143 and rs1800437 in GIPR gene

Primers	Sequence
GIPR5392. R	5’-GGTGGGACAGCATGAGAGATTGTA − 3’
GIPR5392.F	5’-GTTATCTAGCAGCTAACCAGAGATGGA-3’
GIPR0143. R	5’-CCAAGAGTTGGAGACCAGCATGG -3’
GIPR0143.F	5’-CAGTTCCAACAACACTGTCAATCACC-3’
GIPR0143.Nes.R	5’-GTTCCAGTGCACTCCACTCTCAT − 3’
GIPR0143.Nes.F	5’-CAGGCTGGTCTCAAACTCCTG-3’
GIPR00437. R	5’-GCATTCTTGGCATTCTCCTGTCC − 3’
GIPR00437.F	5’-GAAGGAGCTGAGGAAGATCTCAAAGC-3’

F: forward primer, R: Reverse primer, Nes: Nested-PCR

Reactions were performed in a micro-tube with final volume of 25 µl containing 0.2 µg genomic DNA, 0.8 U Hot start Taq DNA polymerase and 1.5 mM MgCl_2_. The temperature cycles for rs34125392 were 94^0^ C for 30 s, 63^0^ C for 45 s and 72^0^ C for 35 s for 30 cycles. The temperature cycles for rs4380143 were 94^0^ C for 30 s, 65^0^ C for 45 s and 72^0^ C for 50 s for 30 cycles. The product of this step was the template for Nested-PCR with new pair of primers (Table 1) to detect rs43800143. The temperature cycles for Nested-PCR were 94^0^ C for 30 s, 62^0^ C for 25 s and 72^0^ C for 35 s for 25 cycles. The temperature cycles for rs1800437 were 94^0^ C for 30 s, 65^0^ C for 45 s and 72^0^ C for 20 s for 30 cycles. Initial incubation period was 5 min at 94^0^ C and a final extension incubation step was set at 72^0^ C for 5 min for all reactions.

Then, the PCR products of rs34125392, rs4380143 and rs1800437 were subjected to digestion with BtsI, FatI and BssSI, respectively (Newengland Biolab; 10U, overnight). The digested PCR products were run on 3% agarose gel and visualized by UV transillumination after DNA green viewer staining.

### Statistical analysis

SPSS v.18 (Chicago) was used for statistical analysis. Normality of the data was checked by Kolmogorov-Smirnov test. Hardy-Weinberg equilibrium was performed to survey allele distribution. Logistic regression was used to survey odds ratios. The numeric data were reported as mean ± Standard Error (SE). Student t test was used to analyze the differences in FBG, HDL-C, LDL-C, TG and age between the patient and healthy groups. Chi square test was applied to investigate the differences of genotype, allele and sex frequencies between the groups. Also the differences of allele and genotype distribution in the men and woman of the both group was assayed by chi square test. Two-way multivariate analysis of variance (Two-way MANOVA) was performed to investigate the effect of independent variables (Polymorphisms) on dependent variables (Biochemical parameters such as FBG, TC, TG, HDL-C and LDL-C).Wilks lambda and Tukey tests were used in Two-way MANOVA. *P* value less than 0.05 was considered to be significant.

## Results

### Characteristics of the study population

The dataset generated and/or analyzed during the current study are available as Supplementary file 1. Two hundred subjects including 100 healthy individuals and 100 T2DM patients were recruited in the study. Characteristics of the study population are summarized in Table 2. Healthy subjects and T2DM patients had a different age (P = 0.029). Distribution of the sex between the both groups was not significantly different (P > 0.05). FBS, LDL-C, TC and TG were statistically different between the groups (P < 0.001). High density lipoprotein cholesterol was not different between T2DM and healthy subjects (P > 0.05).

**Table 2:** Characteristics of study population

Parameters	Control N = 100	T2DM N = 100	P value
**Age(year)**	54 ± 1.23	57.3 ± 0.9	P = 0.029
**HDL-C(mg/dl)**	46.6 ± 2	45.2 ± 3	P > 0.05
**LDL-C(mg/dl)**	90.2 ± 3.02	110.9 ± 4.34	P < 0.001
**TG(mg/dl)**	104.6 ± 3.64	159.6 ± 9.83	P < 0.001
**TC(mg/dl)**	149.9 ± 3.3	186.6 ± 5.33	P < 0.001
**FBS(mg/dl)**	84.7 ± 2.42	166.58 ± 7.09	P < 0.001
**Sex(male/female)**	28/72	40/60	P > 0.05

FBS: fasting blood sugar; HDL-C: high density lipoprotein cholesterol; LDL-C: low density lipoprotein cholesterol; TC: total cholesterol; T2DM: type 2 diabetes mellitus: TG: triacylglycerol

### Genotype/Allele distribution

Distribution of genotypes and allele frequency of rs34125392, rs4380143 and rs1800437 are summarized in Table 3. rs34125392 genotype distribution was statistically different between T2DM and healthy groups(P = 0.043). In addition, distribution of T/- + -/- versus TT was significantly different between the both groups (P = 0.021). Moreover, rs34125392 T/- genotype increased the risk of T2DM (OR = 2.68, 95%CI = 1.203–5.653, P = 0.015). However, allele frequency and distribution of rs34125392 genotype in men and women was not significantly different between the groups (P > 0.05).The effects of the rs34125392 polymorphism on biochemical parameters was investigated by Two-way MANOVA analysis (Table 4). The results showed that the polymorphism and its genotypes had no effect on biochemical parameters (P > 0.05).

**Table 3:** Genotype and allele distribution of rs34125392, rs4380143 and 1,800,437 in study population

Allele/Genotype		Control (n = 100)	T2DM (n = 100)	P value
rs34125392				
Allele	T	97(48.5%)	80(40%)	NS
	-	103(51.5%)	120(60%)	
Genotype	T/T	27(27%)	13(13%)	0.043
	T/-	43(43%)	54(54.9%)	
	-/-	30(30%)	33(33%)	
	T/- + -/-	73	87	0.021
rs4380143				
Allele	T	110(55%)	102(51%)	NS
	C	90(45%)	98(49%)	
Genotype	T/T	23(23%)	16(16%)	NS
	T/C	65(65%)	70(70%)	
	C/C	12(12%)	14(14%)	
	T/C + C/C	77	84	NS
rs1800437				
Allele	G	154(23%)	150(75%)	NS
	C	46(77%)	50(25%)	
Genotype	G/G	7(7%)	5(5%)	NS
	G/C	32(32%	40(40%)	
	C/C	61(61%)	55(55%)	
	G/C + C/C	93	95	NS

**Table 4:** Two-way MANOVA between rs34125392 (T/T versus T/- + -/-), rs4380143 (T/T versus T/C + C/C) and rs1800437 (GG versus G/C + C/C) and biochemical variables in study population

Biochemical variables ( Control versus Patients)	rs34125392 T/T vs. T/- + -/-	rs4380143 T/T vs. T/C + C/C	rs1800437 GG vs. G/C + C/C
	P value	P value	P value
HDL-C	P = 0.617	P = 0.637	P = 0.670
LDL-C	P = 0.719	P = 0.937	P = 0.320
TG	P = 0.444	P = 0.181	P = 0.561
TC	P = 0.720	P = 0.406	P = 0.409
FBS	P = 0.118	P = 0.061	P = 0.592

FBS: fasting blood sugar; HDL-C: high density lipoprotein cholesterol; LDL-C: low density lipoprotein cholesterol; TC: total cholesterol; TG: triacylglycerol; VS: versus

On the other hand, allele frequency and genotype distribution of rs4380143 was not significantly different between healthy and T2DM subjects (P > 0.05). T/C + C/C versus TT were not different between the groups (P > 0.05). In addition, allele frequency and genotypes distribution of rs4380143 was the same in men and women between the groups (P > 0.05). There was no increased risk of rs4380143 for T2DM (P > 0.05). Two-way MANOVA analysis (Table 4) showed that rs4380143 and its genotypes had no effect on biochemical parameters (P > 0.05).

Moreover, rs1800437 genotype distribution and allele frequency was the same in the both groups (P > 0.05). G/C + C/C versus GG were not different between the groups (P > 0.05). We found no increased risk of genotypes and alleles for T2DM (P > 0.05). Two-way MANOVA analysis (Table 4) showed that rs1800437 and its genotypes had no effect on biochemical parameters (P > 0.05). Also the cumulative effects of tested polymorphisms on biochemical parameters were investigated. The findings revealed that the tested polymorphism and their genotypes had no effect on biochemical parameters (Data not shown; P > 0.05).

## Discussion

The main finding of our study was that rs34125392 in GIPR gene is associated with T2DM and this polymorphism increased the risk of the disease. Also multivariate analysis indicated that the tested polymorphisms and their genotypes had no effect on FBG, HDL-C, LDL-C, TC and TG. T2DM is a multifactorial disease which affects many people worldwide [22] and virtually no physician is found that has no patient with T2DM. Gene variations have a strong role in T2DM and the list of the genes that involved in the pathogenesis of the disease is increasing to date [23].

To the best of our knowledge this the first study that investigated rs34125392 and rs4380143 polymorphisms in T2DM. We searched PubMed, Google and dbSNP databases and found no study that included these polymorphisms in their studies. Our findings showed that the distribution of rs34125392 genotypes is different between the healthy and patient group. Also our finding indicated that rs34125392 T/- genotype may increase the risk of T2DM. Taking together, since this polymorphism is located on 5ʹUTR region of GIPR gene and we didn’t measure the expression level of GIPR gene, so the main question is that whether this gene variation could alter gene expression. Skuratovskaia et al. [15] investigated the association of GIPR gene polymorphism with plasma level of mediators which have a role in the regulation of carbohydrate metabolism in obese T2DM patients. They found that GIPR gene expression in adipose tissue of the small intestine mesentery in patients bearing rs2302382 CC and rs8111428 AA genotypes was decreased and this was in relation to increase level of leptin. They claimed that during normal expression plasma concentration of insulin and GIP in subjects bearing rs2302382 polymorphism and rs8111428 AG genotypes were increased. However, we found no increased risk of rs4380143 genotypes in T2DM and the distribution of alleles and genotypes were the same in patients and healthy groups.

Shalaby group [24] studied rs1800437 polymorphism in Egyptian T2DM patients. Their findings indicated that the distribution of C haplotype is statistically higher in patients than controls. They concluded that there is no association between this polymorphism and the risk of T2DM. In contrast to Shalaby group our finding showed that the distribution of rs1800437 genotypes and allele frequency in T2DM patients and healthy subjects were not different. This inconsistency may be related to different races. However, we found that there is no association between rs1800437 genotypes and alleles with T2DM. Jeannine group [25] investigated the association of variants in GIPR gene with impaired glucose homeostasis in obese children and adolescents from Berlin. They found an association between rs1800437 C allele and elevated homeostasis model of insulin resistance values. They concluded that GIPR gene variations are not related to childhood obesity but rs1800437 may have a potential role in glucose homeostasis. Recent studies are focused on the GIPR agonist to improve the extra pancreatic effects of GIP and its role in secretion of insulin. Nicholas group [26] performed a study to clarify whether the increased risk of coronary artery disease (CAD) is mediated via GIPR or is instead the result of linkage disequilibrium (LD) confounding between variants at the GIPR locus. They found that rs1800437 G allele is common among the fasting GIP levels, glycemic traits, and adiposity-related traits and it is independent of CAD and lipid traits.

T2DM patients have a high propensity for multiple comorbidities related to diabetes complications such as cardiovascular disease (CVD). Since there are substantial differences in lipid markers among the study’s baseline characteristics in our study and lipid markers has been proven to be a risk factor of CVD[27], so we analyzed the relation of the tested polymorphism with lipid parameters by Two-way MANOVA. Our findings indicate that there is no association between GIPR polymorphism and lipid parameters.

### Limitation of the study and suggestions

This study has some limitations including low sample size. While this is the first study in Iranian population, we suggest that future studies should be conduct with larger sample size. This was not a linkage study, so we suggest that future studies be conducted with direct haplotyping method to better show the relationship between polymorphism and its influence on biochemical parameters and T2DM.

## Conclusion

our findings indicated that GIPR gene polymorphism is associated with T2DM in Iranian patients. In addition, the results showed that rs34125392 T/- genotype may increase the risk of T2DM. However, the tested polymorphisms had no effect on biochemical parameters. Since rs34125392 polymorphism is located on 5ʹUTR region of GIPR gene, further studies are needed to show that whether genotype variation could alter GIPR phenotype. Future studies with larger sample size in other populations are recommended to show the ethnical relation of these polymorphisms to T2DM. Our sample size was relatively small so our findings should interpret with caution.

## Electronic supplementary material

Below is the link to the electronic supplementary material.

Supplementary file 1. The data sete analysed during the study to investigate genotype and allele frequency of rs34125392, rs4380143 and rs1800437 in GIPR gene

## Data Availability

The dataset generated and/or analyzed during the current study are available as Supplementary file 1.

## List of abbreviations

CAD: Coronary artery disease
DM: Diabetes mellitus
FBG: Fasting blood glucose
GIPR: Gastric inhibitory polypeptide receptor
GIP: Gastric inhibitory polypeptide
HDL-C: High density lipoprotein cholesterol
LD: Linkage disequilibrium
LDL-C: Low density lipoprotein cholesterol
LPL: Lipoprotein lipase
MAF: Minor allele frequency
TC: Total cholesterol
T2DM: Type 2 diabetes mellitus
TG: Triacylglycerol
